# Perceptions of overactive bladder treatments among women, caregivers, and healthcare professionals: a systematic review and meta-synthesis

**DOI:** 10.3389/fgwh.2026.1974337

**Published:** 2026-10-28

**Authors:** Maria Patricia Roman, Dan Mihu, Denisa Rus, Nicole Stephanie Olaru, Carmen Mihaela Mihu, Răzvan Ciortea, Stergios K. Doumouchtsis, Andrei Roman, Carmen Elena Bucuri, Cristina Mihaela Ormindean, Viorela Elena Suciu, Ionel Daniel Nati, Andrei Mihai Măluțan

**Affiliations:** 1 2nd Obstetrics and Gynecology Clinical Section, Cluj County Emergency Clinical Hospital, Cluj-Napoca, Romania; 2 2nd Discipline of Obstetrics and Gynecology, Mother and Child Department, “Iuliu Hațieganu” University of Medicine and Pharmacy, Cluj-Napoca, Romania; 3 Discipline of Histology, Department of Morpho-functional Sciences, Iuliu Haţieganu University of Medicine and Pharmacy, Cluj-Napoca, Romania; 4 Department of Obstetrics and Gynecology, Epsom and St. Helier University Hospitals NHS Trust, Epsom, United Kingdom; 5 St. George’s University of London, London, United Kingdom; 6 Laboratory of Experimental Surgery and Surgical Research “N.S. Christeas,” National and Kapodistrian University of Athens, Medical School, Athens, Greece; 7 School of Medicine, American University of the Caribbean, Cupecoy, Sint Maarten; 8 School of Medicine, Ross University, Miramar, FL, United States; 9 Radiology Department, Prof. Dr. Ion Chiricuţă Institute of Oncology, Cluj-Napoca, Romania; 10 Discipline of Radiology and Imaging, Surgical Specialties Department, Iuliu Hațieganu University of Medicine and Pharmacy, Cluj-Napoca, Romania; 11 Gynecology Compartment, Military Emergency Hospital “Dr. Constantin Papilian”, Cluj-Napoca, Romania

**Keywords:** lived experience, meta-synthesis, perceptions, qualitative evidence synthesis, qualitative research, refractory overactive bladder, treatment, women

## Abstract

**Introduction:**

Overactive bladder (OAB) is a common condition that substantially impairs quality of life yet remains underreported and undertreated. Understanding how women, caregivers, and healthcare professionals (HCPs) perceive OAB treatments may help improve patient-centered care. This systematic review and meta-synthesis aims to synthesize qualitative evidence on experiences and perceptions of OAB treatment.

**Methods:**

MEDLINE, Scopus, Google Scholar and CINAHL were searched from inception to January 2026 for qualitative studies exploring treatment experiences among women with OAB, their caregivers, and HCPs. Three reviewers independently screened studies, extracted data, and performed line-by-line coding. Data were synthesized using thematic synthesis. Methodological quality was assessed using the Critical Appraisal Skills Programme (CASP) checklist. A GRADE-CERQual assessment was performed to evaluate confidence in the synthesized qualitative findings.

**Results:**

Thirteen qualitative studies were included. Forty-five codes were identified and synthesized into five analytical themes: (1) the burden of treatment vs. the burden of illness, reflecting the tension between symptom burden and treatment-related challenges; (2) erosion of trust in the therapeutic alliance, driven by poor communication and unmet expectations; (3) the knowledge gap and desire for agency, highlighting unmet informational needs and preferences for shared decision-making; (4) psychosocial barriers to treatment engagement, encompassing stigma, embarrassment, isolation, and emotional distress; and (5) preference for low-burden treatments and resistance to behavioural change, reflecting preferences for convenient therapies and difficulties sustaining long-term behavioural interventions.

**Conclusions:**

Experiences of OAB treatment extend beyond symptom control and are shaped by psychosocial burden, healthcare interactions, treatment expectations, and the need for autonomy. Patient-centered communication, shared decision-making, and individualized support may improve treatment engagement, satisfaction, and adherence.

**Systematic Review Registration:**

https://www.crd.york.ac.uk/PROSPERO/view/CRD420261289760, Identifier: CRD420261289760.

## 1 Introduction

Overactive bladder syndrome (OAB) is characterized by urinary urgency, typically accompanied by increased daytime frequency and nocturia, with or without urgency urinary incontinence (1, 2). This symptom complex is highly prevalent, affecting approximately twice as many women as men (3, 4). Reported prevalence in women ranges widely from 22% to 60%, with rates increasing progressively with age (5, 6).

This condition negatively impacts quality of life and may contribute to social isolation. OAB is also associated with substantial psychological and economic burden and, in older women, may be linked to increased morbidity and mortality.

Management follows a stepwise approach, progressing from conservative (behavioral and lifestyle interventions) to pharmacologic therapy and, when indicated, minimally invasive procedures (7, 8). Despite this range of options, OAB remains underreported and consequently undertreated in clinical practice (9, 10). Epidemiological data suggest that while up to 60% of women seek medical attention, an even smaller proportion (47%) receive actual treatment (11–13).

Addressing this gap requires moving beyond clinical efficacy to consider patient-related barriers such as stigma, misperceptions about aging, and inadequate patient-provider communication. Women's perceptions of treatment are shaped by multiple factors, including educational level, family support, and the quality of guidance from HCPs, yet their perspectives remain incompletely integrated into treatment decision-making.

Existing qualitative studies have largely explored the perspectives of individual stakeholder groups in isolation, focusing separately on women living with OAB, HCPs, or family caregivers. Consequently, opportunities to identify shared priorities, divergent expectations, and interactions between these perspectives have been limited. Integrating the experiences of multiple stakeholders within a single meta-synthesis may provide a more comprehensive understanding of treatment engagement, communication, and decision-making across the OAB care pathway.

Moreover, although individual qualitative studies have substantially advanced understanding of patients' experiences with OAB treatment, their findings are typically shaped by specific interventions, healthcare settings, and sociocultural contexts. A meta-synthesis does more than simply aggregate data; it offers a framework to critically reinterpret findings across diverse studies and distil higher-order analytical themes and conceptual frameworks that extend beyond any single dataset. By identifying common patterns, tensions, and relationships between experiences, meta-synthesis can produce novel interpretive knowledge capable of informing patient-centred care, shared decision-making, core outcome set development, and clinical guideline recommendations.

Building on this rationale, this systematic review and meta-synthesis aims to explore womens', caregivers', and healthcare professionals' perceptions and experiences of OAB treatment, with particular attention to treatment failure, persistence, barriers to treatment escalation, and third-line therapies. The findings are intended to identify patient- and stakeholder-relevant concepts that may inform future research, outcome measurement, and guideline development following appropriate consensus and validation processes.

## 2 Materials and methods

### 2.1 Study registration

This study was registered in the International Prospective Register of Systematic Reviews (PROSPERO; registration number CRD420261289760) and was performed in accordance to the Enhancing Transparency in Reporting the Synthesis of Qualitative Research (ENTREQ) reporting guidelines (14) (Supplementary Material 1).

### 2.2 Search strategy and study selection

A predefined search strategy was developed for this meta-synthesis to identify all available primary qualitative studies exploring the lived experiences and perspectives of women with OAB, their caregivers, and HCPs involved in the treatment and management of this condition. Using a structured combination of keywords (Supplementary Material 2), this approach systematically searched the MEDLINE, Scopus, Google Scholar and CINAHL databases from their inception up to January 2026. For Google Scholar, the first 100 results were screened, in accordance with recommended practice, as results beyond this threshold tend to yield fewer relevant studies and a higher degree of duplication (15).

Specific inclusion criteria were established to ensure both relevance and methodological rigor. Eligible studies included women with OAB, as well as family caregivers and healthcare professionals involved in their care. Primary studies including male and female patient populations were eligible only when findings attributable to women could be identified and extracted separately. For such studies, only findings attributable to female participants were included in the qualitative synthesis. Additionally, studies were eligible if they provided qualitative evidence relevant to OAB treatment experiences, including treatment response or failure, persistence and discontinuation, treatment burden, treatment decision-making, or progression to third line therapies in refractory cases, employed a qualitative methodology and were published in English in peer-reviewed journals. The search strategy was intentionally enriched for refractory OAB and third-line treatments, reflecting the review's particular interest in difficult-to-treat disease and treatment escalation rather than the full spectrum of OAB treatment.

The phenomenon of interest comprised experiences, perceptions, attitudes, preferences, and barriers related to OAB treatment, including treatment response or failure, adherence and discontinuation, treatment burden, treatment decision-making, and progression to third-line therapies. Primary studies using qualitative methods, including interviews, focus groups, phenomenological approaches, grounded theory, and qualitative components of mixed-methods studies, were eligible when qualitative findings could be extracted separately.

Studies addressing OAB symptoms without treatment-related experiences, purely quantitative studies, non-peer-reviewed publications, and non-English-language publications were excluded.

Study selection followed a systematic, two-stage assessment process to ensure rigorous and unbiased inclusion. In the first stage, three researchers (MPR, DR, NSO) independently screened the titles and abstracts of all identified records against the predefined inclusion criteria. Records meeting these criteria proceeded to a full-text review, during which the same three reviewers independently assessed each study in greater detail. Discrepancies at any stage were resolved through discussion within the research team. This three-reviewer approach minimized bias and strengthened the reliability of the selection process.

To identify additional eligible studies, snowballing was conducted by examining the reference lists of included studies and relevant reviews. The complete selection process was documented using a PRISMA flow diagram Figure 1, which records the number of studies screened, excluded, and finally included in the synthesis.

**Figure 1:**
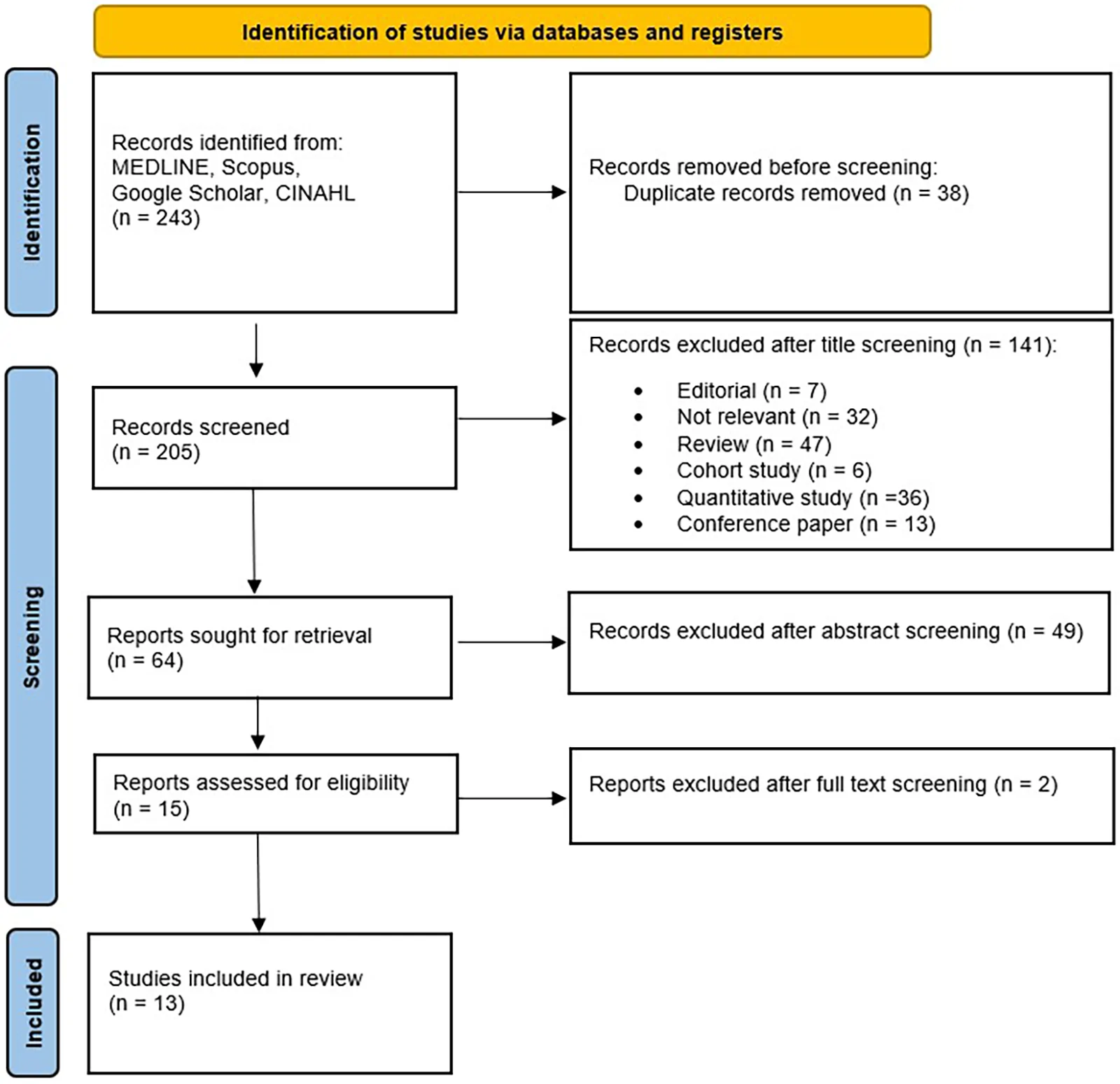
Prisma flowchart.

### 2.3 Data extraction and synthesis

A modified Cochrane data collection form for intervention reviews (non-RCTs) (16) was used for data extraction (Supplementary Material 3). The following data were recorded: aim of the study, treatment type, geographic location, sample size, source of study sample, participant's details, data analysis method, emerging themes and data saturation.

Line-by-line coding was undertaken by three researchers (MPR, DR, NSO), all trained in qualitative research, to ensure analytical rigor and minimize interpretive bias. The software used for qualitative data analyses was Atlas.ti (version 26) (17).

All studies were coded independently, and the resulting codes were compared and centrally consolidated through iterative discussions. Discrepancies were resolved through consensus, with continuous reference to the relevant participants' quotations reported in the included studies. Where reviewers had generated identical codes, those codes were retained; where codes reflected similar meanings, they were merged under a common label; and where disagreement arose, they were discussed in depth and re-examined against the source material. Code frequency was calculated as the total number of coded segments assigned to each code across the included studies. These frequencies were used descriptively to characterize the coding dataset and were not interpreted as measures of prevalence, stakeholder representation, or thematic importance.

Agreed codes were subsequently compared for conceptual similarity and grouped into broader categories representing related ideas across the included studies. These categories formed the descriptive themes, which summarized recurring patterns in participants' accounts while remaining close to the original data.

The synthesis followed the thematic synthesis framework described by Thomas and Harden (18), comprising line-by-line coding, development of descriptive themes, and generation of higher-order analytical themes. The inductive and iterative coding and theme-development process was additionally informed by the principles of thematic analysis described by Braun and Clarke (19). During this stage, descriptive themes were systematically compared, merged, or reorganized by exploring relationships between them and asking what broader concept or explanatory construct accounted for the observed patterns. The resulting analytical themes therefore represented higher-order interpretations that integrated multiple descriptive themes into overarching concepts explaining participants' treatment experiences. Throughout this process, alternative thematic structures were considered and discussed among the reviewers, and discrepant or contradictory findings were actively examined to challenge emerging interpretations and strengthen the credibility of the final analytical framework.

### 2.4 Risk of bias assessment

To enhance the methodological rigor of qualitative synthesis' findings, risk of bias assessment of the primary studies is recommended. Provided that there is no international consensus on a single best tool for critical appraisal of qualitative studies, the Critical Appraisal Skills Programme (CASP) (20), endorsed by the Cochrane Qualitative and Implementation Methods Group (21), was used for the purpose of this study. This tool provides a systematic, 10-items framework with a focus on study design, methodology, data analysis, and ethical considerations to determine the trustworthiness of qualitative findings. Each item on the CASP Qualitative Studies Checklist was rated using one of three outcomes: “Yes” (criterion fully met), “Can't tell” (insufficient detail or transparency to make a clear judgment as to whether the criterion was met), or “No” (criterion not met).

The review team comprised clinicians and researchers with expertise in urogynecology, women's health, and qualitative research. The team's clinical backgrounds were recognized as having the potential to influence the analytical process by sensitizing interpretation to issues such as treatment adherence, patient–clinician communication, and shared decision-making, while potentially placing less emphasis on broader social or cultural dimensions of participants' experiences. All studies were appraised independently by the three assessors (MPR, DR, NSO) using the CASP tool, and discrepancies were resolved through consensus discussions grounded in the study data and methodological criteria, rather than clinical assumptions.

To enhance reflexivity, an inductive thematic synthesis approach was adopted, whereby codes and themes were derived from the primary data rather than from predefined clinical frameworks. Throughout the analysis, coding decisions and emerging interpretations were discussed iteratively among the reviewers to identify and challenge underlying assumptions, particularly when participants' perspectives diverged from prevailing clinical practice or professional expectations. Original participant quotations were revisited during theme development to ensure that the analytical themes remained grounded in the source data and reflected the diversity of experiences across the included studies. This reflexive process sought to leverage the team's clinical expertise while minimizing the influence of disciplinary preconceptions on the interpretation of the findings.

Confidence in the synthesized qualitative findings was assessed by three reviewers (MPR, DR, NSO) using the GRADE-CERQual (Confidence in the Evidence from Reviews of Qualitative Research) approach (22). Any disagreements between the reviewers were resolved through consensus discussions within the team. Each review finding was evaluated across four components: methodological limitations of the contributing studies, coherence of the finding, adequacy of the supporting data, and relevance to the review question. Based on these assessments, an overall level of confidence (high, moderate, low, or very low) was assigned to each synthesized finding.

## 3 Results

This meta-synthesis included 13 primary qualitative studies (23–35) exploring experiences of OAB treatment among women, their carers, and HCPs. Table 1 summarizes the methodological and contextual characteristics of the included studies, including geographical setting, participant characteristics, sample source, sample size, data collection methods, analytical approaches, investigated treatments, and reporting of data saturation.

**Table 1:** Characteristics of primary studies.

Study	Country	Setting/sample source	Participants	Sample size	Data collection	Data analysis	Investigated treatment	Data saturation
Sitto et al. (29)	USA	Web-based survey recruitment	Patients and physicians	32	Semi-structured interviews	Inductive thematic analysis	Not mentioned	Achieved
Daly et al. (25)	UK	Outpatient urogynaecology clinics	Women with OAB	16	Individual interviews	Reflexive thematic analysis	Transcutaneous Tibial Nerve Stimulation (TTNS)	Not specified
Turell et al. (35)	USA	Digital marketing/social media	HCPs involved in OAB educational program	20	Interviews	Constant comparative method	N/A	Not specified
Davenport et al. (26)	USA	Urogynaecology/urology departments	Women with refractory OAB	56	Interviews	Grounded theory	Third-line therapies	Achieved
Ali et al. (23)	UK	Primary care patient lists	Adults with OAB	18	Semi-structured interviews	Thematic analysis	Mirabegron, anticholinergics, botulinum toxin	Achieved
Kinsey et al. (27)	UK	Urology outpatient clinic	Women receiving anticholinergics	5	Individual interviews	Thematic analysis	Anticholinergics	Not specified
Siu (32)	Hong Kong	Patient self-help group	Adults with self-reported OAB	30	Semi-structured interviews	Phenomenology	Behavioral therapy/anticholinergics	Not specified
Maguire et al. (28)	UK	Clinical trial participants	Women with detrusor overactivity	15	Telephone interviews	Grounded theory	Conservative strategies, anticholinergics, botulinum toxin	Achieved
Siu (31)^a^	Hong Kong	Caregiver referral by patients	Family caregivers of patients with OAB	35	Semi-structured interviews	Phenomenology	N/A	Not specified
Siu (33)^b^	Hong Kong	Caregiver referrals	Primary care doctors	35	Semi-structured interviews	Phenomenology	N/A	Not specified
Siu (30)	Hong Kong	OAB self-help group	Women with OAB	30	Semi-structured interviews	Phenomenology	Behavioral, pharmacological, instillations, complementary and alternative medicine therapies	Not specified
Anger et al. (24)	USA	Female urology clinics	Women with OAB	33	Focus groups	Grounded theory	Conservative treatment, anticholinergics, sacral neuromodulation (SNM), botulinum toxin	Achieved
Smith et al. (34)	USA	Female urology specialty clinics	Women with OAB	33	Focus groups	Grounded theory	Pelvic floor therapy, anticholinergics, SNM, botulinum toxin	Not specified

a Refers to the following study: Coping with patients suffering from overactive bladder: experiences of family caregivers in Hong Kong.

b Refers to the following study: Communicating with mismatch and tension: treatment provision experiences of primary care doctors treating patients with overactive bladder in Hong Kong.

Table 2 presents the aims of the included studies together with their main emerging themes, providing an overview of the qualitative concepts contributing to thematic synthesis.

**Table 2:** Aims of included studies and their emerging themes.

Study	Study aim	Emerging themes
Sitto et al. (29)	To compare patient and physician perceptions regarding OAB treatment.	Treatment inaccessibility; discordant educational perceptions; differing expectations of outcomes.
Daly et al. (25)	To explore women's experiences with TTNS for OAB.	Everyday impact of bladder control loss; self-management versus HCP-led treatment; treatment preferences.
Turell et al. (35)	To explore HCPs’ perspectives on OAB recognition and management.	Taking OAB seriously; variation in therapeutic approaches; importance of patient motivation and education.
Davenport et al. (26)	To investigate women's experiences and attitudes toward third-line OAB therapies.	Treatment delay; educational needs; attitudes toward treatment; office-related barriers.
Ali et al. (23)	To assess patient experiences with pharmacological OAB therapies.	Support with treatment; treatment discontinuation; unmet efficacy and tolerability expectations.
Kinsey et al. (27)	To examine patient experiences with anticholinergic medication for OAB.	Hidden burden of OAB; impact of treatment; OAB as central life concern.
Siu et al. (32)	To examine the lived experiences of individuals with OAB.	Cultural stereotypes; denial; concealment; resignation; self-seclusion.
Maguire et al. (28)	To evaluate participants’ experiences within a clinical trial for OAB treatment.	Impact on quality of life; conservative management experiences; participation in RCTs.
Siu (31)^a^	To explore the experiences of family caregivers of patients with OAB.	Powerlessness; helplessness; confusion; social withdrawal; caregiver burden.
Siu (33)^b^	To explore primary care doctors burden associated with OAB.	Helplessness; confusion; guilt; grievances; social withdrawal.
Siu et al. (30)	To explore illness experiences among women with OAB.	Embarrassment; shame; frustration; helplessness; resignation; self-seclusion.
Anger et al. (24)	To investigate coping strategies and treatment experiences among women with OAB.	Self-blame; search for cause and cure; personalized symptom management; daily triggers.
Smith et al. (34)	To investigate misconceptions and miscommunication related to OAB.	Misunderstanding of OAB etiology; confusion regarding diagnostics and treatment; patient-provider miscommunication.

a Refers to the following study: Coping with patients suffering from overactive bladder: experiences of family caregivers in Hong Kong.

b Refers to the following study: Communicating with mismatch and tension: treatment provision experiences of primary care doctors treating patients with overactive bladder in Hong Kong.

### 3.1 Thematic synthesis

After independent line-by-line coding by three researchers and subsequent centralization of the results, 45 distinct codes were identified across the included studies. The emerging codes, as well as their frequency are presented in Figure 2. In this meta-synthesis, code frequency indicates how often a concept was expressed across all primary study data. The frequency of each code varied considerably, from concepts mentioned only twice to those occurring 40 times across the data set. Although code frequencies are presented to illustrate the recurrence of concepts across the included studies, they should not be interpreted as reflecting the relative importance of individual findings. In qualitative synthesis, analytical significance is determined by the interpretive contribution of a concept rather than the number of times it is reported. Theme development therefore relied on the meaning, context, and explanatory value of the coded data, rather than on frequency alone.

**Figure 2:**
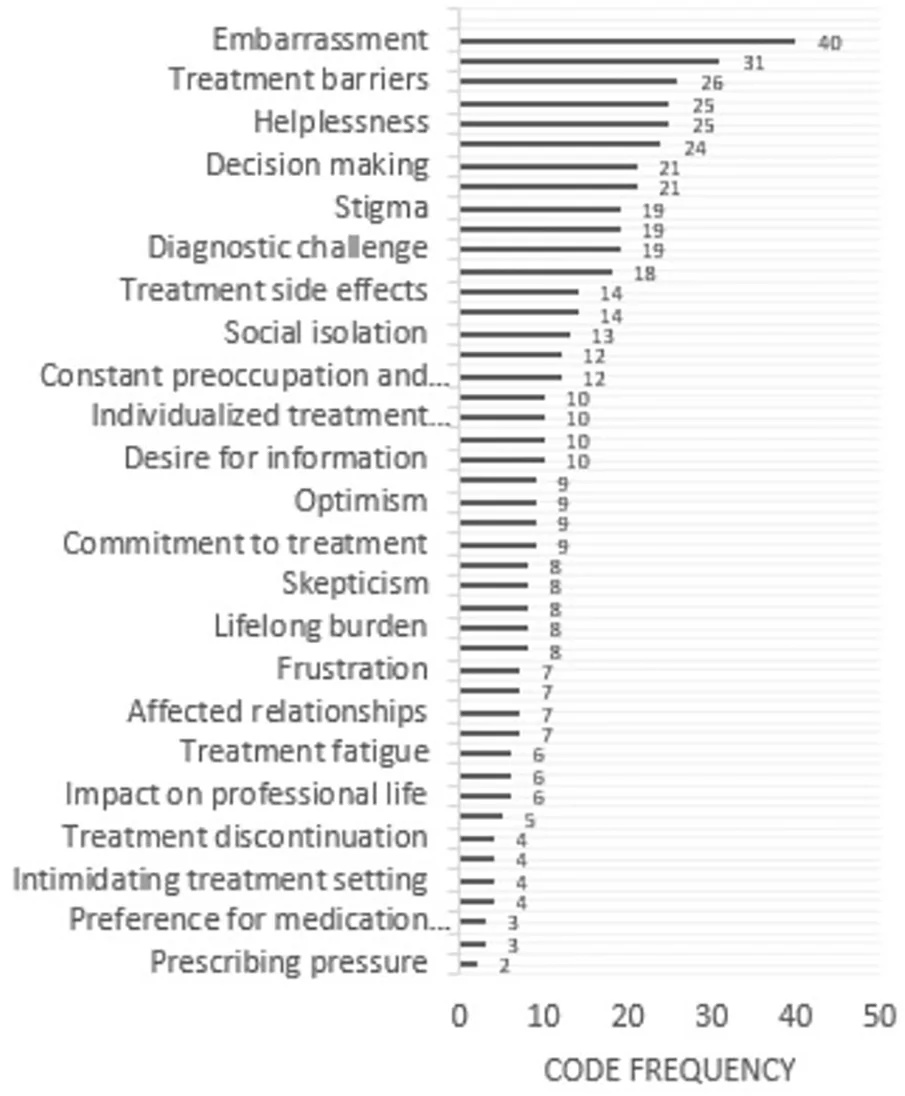
Frequency of codes across the included studies.

The thematic synthesis generated a hierarchical interpretive framework consisting of descriptive themes that converged into higher-order analytical themes. Through iterative comparison and interpretive synthesis of the included studies, participants' experiences of OAB treatment were organized into five overarching analytical themes reflecting treatment burden, healthcare interactions, knowledge and decision-making, psychosocial experiences, and treatment preferences. A comprehensive visual representation of the theme development process, illustrating the descriptive themes that converged into higher-order analytical themes, is presented in Figure 3.

**Figure 3:**
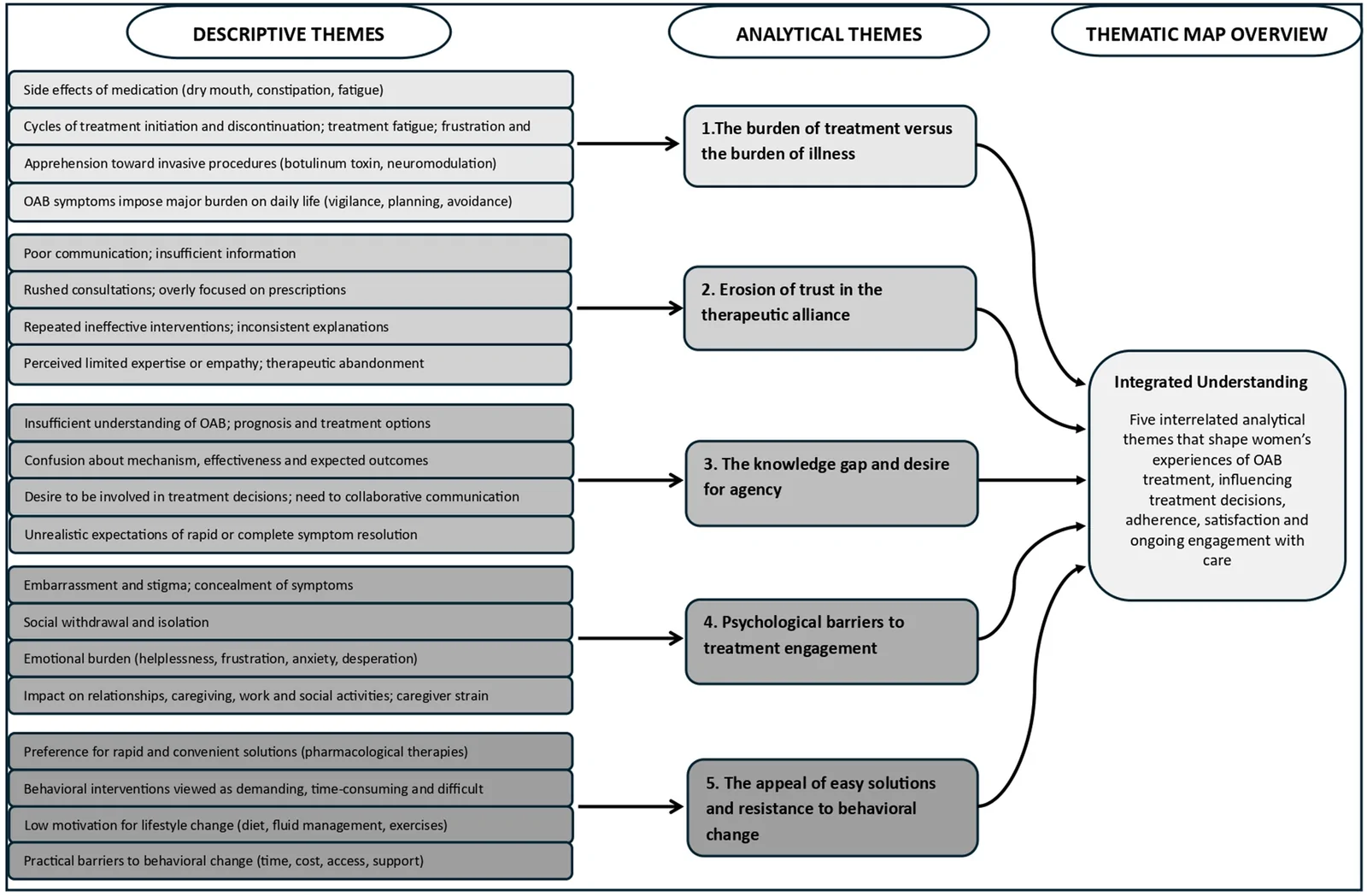
Thematic map illustrating how descriptive themes (first-order findings) converge into five analytical themes (higher-order interpretations) in this synthesis of participants’ experiences of OAB treatments.

#### 3.1.1 Descriptive themes contributing to analytical theme 1: the burden of treatment vs. the burden of illness

Participants frequently described treatment side effects as a major barrier to sustained engagement with care. Pharmacological therapies were commonly associated with adverse effects such as dry mouth, constipation, fatigue, and concerns regarding long-term medication use. Repeated cycles of treatment initiation and discontinuation contributed to treatment fatigue and reinforced feelings of frustration and helplessness. Alongside medication-related concerns, women also expressed apprehension toward invasive procedures, including botulinum toxin injections and neuromodulation therapies. Fear of pain, uncertainty regarding efficacy, and intimidating treatment settings contributed to reluctance to pursue advanced interventions.

At the same time, OAB symptoms themselves imposed a substantial burden on everyday life. Participants described persistent vigilance regarding bladder symptoms, constant planning around toilet access, avoidance of social situations, and disruption of professional and interpersonal activities. Many women perceived OAB as a chronic and lifelong condition associated with ongoing reductions in quality of life.

These descriptive themes converged into the analytical theme “The burden of treatment vs. the burden of illness,” which captured the tension between the debilitating nature of OAB symptoms, and the perceived burden associated with available treatment options.

#### 3.1.2 Descriptive themes contributing to analytical theme 2: erosion of trust in the therapeutic alliance

Many participants described negative healthcare experiences characterized by poor communication, insufficient information, and unmet expectations regarding treatment outcomes. Consultations were frequently perceived as rushed, overly focused on prescriptions, or lacking meaningful discussion regarding patient concerns and preferences. Participants also reported frustration with repeated ineffective interventions and inconsistent explanations regarding diagnosis and management.

Women often perceived HCPs as having limited expertise or empathy regarding OAB management. Diagnostic uncertainty and recurrent treatment failures contributed to doubts regarding clinical competence and intensified feelings of therapeutic abandonment. Over time, repeated negative interactions fostered mistrust toward HCP and skepticism regarding future treatments. In some cases, participants sought alternative therapies or disengaged from healthcare services altogether.

Together, these descriptive themes contributed to the analytical theme “Erosion of trust in the therapeutic alliance,” emphasizing the central role of communication and therapeutic alliance in treatment adherence and healthcare engagement.

#### 3.1.3 Descriptive themes contributing to analytical theme 3: the knowledge gap and desire for agency

Participants consistently reported insufficient understanding of OAB pathophysiology, prognosis, and available therapeutic options. Many women expressed confusion regarding the mechanisms, effectiveness, and expected outcomes of treatment, contributing to uncertainty and anxiety throughout the treatment journey.

Alongside these knowledge gaps, participants expressed a strong desire to participate actively in treatment decisions. Individualized support, collaborative communication, and access to understandable information increased feelings of empowerment and facilitated engagement with care. However, unrealistic expectations regarding rapid or complete symptom resolution often resulted in disappointment when treatments provided only partial or delayed improvement.

These descriptive themes converged into the analytical theme “The knowledge gap and desire for agency,” highlighting the importance of information, shared decision-making, and individualized support in shaping treatment experiences.

#### 3.1.4 Descriptive themes contributing to analytical theme 4: psychosocial barriers to treatment engagement

Embarrassment and stigma emerged as pervasive experiences across studies. Participants frequently concealed their symptoms from family members, friends, and colleagues due to fear of judgment, humiliation, and social rejection. This concealment often contributed to social withdrawal and isolation.

The emotional burden associated with OAB was substantial. Women described feelings of helplessness, frustration, anxiety, and desperation related to the chronic and unpredictable nature of symptoms. OAB also negatively affected relationships, caregiving responsibilities, occupational functioning, and participation in social activities. Caregivers themselves reported emotional strain and disruption to daily routines.

Collectively, these descriptive themes formed the analytical theme “Psychosocial barriers to treatment engagement,” illustrating how emotional distress and stigma shaped both treatment-seeking behaviors and ongoing engagement with care. These findings are strongly corroborated by recent qualitative work by Brodsky et al. (36), who identified isolation, social stigma, and embarrassment as central themes in the lived experience of women with OAB. Notably, patients in their study also reported feeling “invalidated and dismissed” by physicians, further compounding the psychosocial burden of the condition.

#### 3.1.5 Descriptive themes contributing to analytical theme 5: preference for low-burden treatments and resistance to behavioral change

Many participants favored rapid and convenient treatment options, particularly pharmacological therapies, over behavioral interventions perceived as demanding or time-consuming. Convenience strongly influenced treatment acceptability, and some women expressed frustration with therapies requiring sustained lifestyle modification or long-term commitment.

Behavioral interventions such as bladder training, pelvic floor exercises, and dietary modifications were often difficult to integrate into everyday life. As a result, some participants sought complementary or alternative therapies when conventional approaches failed to meet expectations. Positive treatment experiences and gradual acceptance of diagnosis facilitated longer-term adaptation and improved commitment to management strategies.

These descriptive themes converged into the analytical theme “Preference for low-burden treatments and resistance to behavioral change,” reflecting the tension between the desire for immediate symptom relief and the sustained effort required for effective long-term management.

Supplementary Material 4 provides a detailed overview of the analytical themes, including relevant participant quotations and the studies contributing to each code.

##### Convergence and divergence across stakeholder groups

Across the five themes, several perspectives were shared among women, caregivers, and healthcare professionals, particularly regarding the burden of persistent symptoms, limitations of available treatments, and the importance of effective communication and realistic expectations. Although some perspectives largely converged, each stakeholder group highlighted different aspects of the treatment experience: women focused more strongly on the personal burden, acceptability, and perceived effectiveness of treatment; caregivers highlighted the practical and relational consequences of persistent symptoms; and healthcare professionals emphasized adherence, treatment expectations, and barriers to treatment progression. These perspectives were therefore complementary rather than uniformly concordant and provided various standpoints of the same treatment pathway.

### 3.2 Risk of bias assessment

The CASP appraisal identified generally clear research aims and appropriate qualitative methodologies across the included studies. Methodological concerns were primarily related to insufficient reporting of researcher–participant relationships and reflexivity, which represented the most frequently underreported domain. Additional concerns in some studies involved recruitment strategies, data collection procedures, and ethical considerations. These limitations varied across studies and primarily reflected incomplete reporting rather than consistently identified methodological weaknesses. Study-level assessments for each CASP domain are presented in Table 3.

**Table 3:** Critical appraisal skills program assessments.

Article	Section A: Are the results valid?				Section B: What are the results				Section C: Will the results help locally?	
	Was there a clear statement of the aims of the research?	Is a qualitative methodology appropriate?	Was the research design appropriate to address the aims of the research?	Was the recruitment strategy appropriate to the aims of the research?	Was the data collected in a way that addressed the research issue?	Has the relationship between researchers and participants been adequately considered?	Have ethical issues been taken intoconsideration?	Was the data analysis sufficiently rigorous?	Is there a clear statement of findings?	Is the research valuable?
Sitto et al. (29)	y	y	y	y	y	n	ct	y	y	y
Daly et al. (25)	y	y	y	y	ct	ct	ct	y	y	y
Turell et al. (35)	y	y	y	y	ct	ct	y	y	y	y
Davenport et al. (26)	y	y	y	y	y	ct	y	y	y	y
Ali et al. (23)	y	y	y	y	y	y	y	y	y	y
Kinsey et al. (27)	y	y	y	n	ct	n	y	y	y	y
Siu et al. (32)	y	y	y	y	ct	y	ct	y	y	y
Maguire et al. (28)	y	y	y	y	y	y	y	y	y	y
Siu (31)^a^	y	y	y	y	ct	y	y	y	y	y
Siu (33)^b^	y	y	y	ct	y	y	y	y	y	y
Siu et al. (30)	y	y	y	y	y	y	y	y	y	y
Anger et al. (24)	y	y	y	ct	y	n	ct	y	y	y
Smith et al. (34)	y	y	y	ct	ct	n	ct	y	y	y

Legend: y, yes; n, no; ct, can't tell.

a Refers to the following study: Coping with patients suffering from overactive bladder: experiences of family caregivers in Hong Kong.

b Refers to the following study: Communicating with mismatch and tension: treatment provision experiences of primary care doctors treating patients with overactive bladder in Hong Kong.

The GRADE-CERQual assessment demonstrated varying levels of confidence across the synthesized findings, reflecting differences in methodological limitations, coherence, adequacy, and relevance of the contributing evidence. Detailed judgments and overall confidence ratings for each synthesized finding are presented in Table 4.

**Table 4:** GRADE-CERQual summary of qualitative review findings.

#	Summarised review finding	GRADE-CERQual Assessment of confidence	Explanation of GRADE-CERQual Assessment	References
1	The burden of treatment versus the burden of illness: the tension between the debilitating nature of OAB symptoms, and the perceived burden associated with available treatment options	Moderate confidence	Moderate concerns regarding methodological limitations, Minor concerns regarding coherence, No/Very minor concerns regarding adequacy, and Minor concerns regarding relevance	Siu JY (32); Davenport A et al. (26); Anger JT et al. (24); Sitto HM et al. (29); Daly CME et al. (25); Kinsey et al. (27); Maguire et al. (28)
2	Erosion of trust in the therapeutic relationship: the central role of communication and therapeutic alliance in treatment adherence and healthcare engagement	High confidence	Minor concerns regarding methodological limitations, No/Very minor concerns regarding coherence, No/Very minor concerns regarding adequacy, and Minor concerns regarding relevance	Siu JY (33); Siu JY (32); Siu JY (31); Turell W et al. (35); Davenport A et al. (26); Sitto HM et al. (29); Smith AL et al. (34); Maguire et al. (28)
3	The knowledge gap and desire for agency: the importance of information, shared decision-making, and individualized support in shaping treatment experiences	Moderate confidence	Minor concerns regarding methodological limitations, Minor concerns regarding coherence, No/Very minor concerns regarding adequacy, and Minor concerns regarding relevance	Siu JY (30); Turell W et al. (35); Davenport A et al. (26); Sitto HM et al. (29); Kinsey et al. (27); Maguire et al. (28)
4	Psychosocial barriers to treatment engagement: emotional distress and stigma shape both treatment-seeking behaviors and ongoing engagement with care	Moderate confidence	No/Very minor concerns regarding methodological limitations, Minor concerns regarding coherence, Minor concerns regarding adequacy, and Minor concerns regarding relevance	Siu JY (33); Siu JY (32); Siu JY (30); Maguire et al. (28)
5	The appeal of easy solutions and resistance to behavioral change: the tension between the desire for immediate symptom relief and the sustained effort required for effective long-term management	Moderate confidence	Minor concerns regarding methodological limitations, No/Very minor concerns regarding coherence, No/Very minor concerns regarding adequacy, and Moderate concerns regarding relevance	Siu JY (32); Ali M et al. 2019; Turell W et al. (35); Sitto HM et al. (29); Daly CME et al. (25); Kinsey et al. (27)

## 4 Discussion

### 4.1 Main findings

This meta-synthesis provides a comprehensive interpretation of how women, their carers and HCPs experience and perceive treatments for OAB. Across 13 primary qualitative studies, participants consistently described OAB treatment as extending beyond symptom control alone, encompassing emotional burden, social stigma, healthcare interactions, treatment accessibility, and the need for individualized support.

Some variation in emphasis was observed across geographical settings, with studies conducted in Hong Kong more frequently reporting stigma and concealment, while studies from the United States and United Kingdom more often emphasized healthcare access and treatment decision-making. These patterns should be interpreted cautiously and cannot be attributed to cultural differences alone. In particular, several Hong Kong studies originated from the same research group, and differences across studies may also reflect sampling strategies, research questions, interview approaches, healthcare settings, and analytical perspectives. Further research across diverse cultural and healthcare contexts is needed to determine whether these patterns represent meaningful sociocultural differences.

A central finding of this meta-synthesis was the tension between the burden imposed by OAB symptoms and the burden associated with available treatments. Women frequently described treatment side effects, uncertainty regarding efficacy, and apprehension toward invasive interventions as major barriers to sustained treatment engagement. At the same time, OAB symptoms themselves profoundly disrupt everyday life, contributing to hypervigilance, social withdrawal, reduced quality of life, and ongoing emotional distress. These findings align with previous quantitative literature demonstrating that treatment adherence in OAB remains suboptimal despite the availability of multiple therapeutic options. The present synthesis extends these findings by illustrating how treatment discontinuation is often not solely related to efficacy, but rather to the cumulative emotional, physical, and psychosocial burden associated with both the condition and its management.

Another important finding was the central role of therapeutic alliance in shaping treatment experiences. Participants frequently described poor communication, rushed consultations, insufficient information, and perceived lack of empathy or expertise from HCPs. Recurrent treatment failures and diagnostic uncertainty further contributed to mistrust and disengagement from care. These findings reinforce growing recognition that communication quality and shared decision-making are essential components of effective pelvic floor care. While a purely biomedical model tends to measure success through symptom reduction alone, participants framed positive outcomes differently, emphasising instead the value of being heard, validated, and included as active partners in their own care. This aligns with the growing recognition that OAB is a “preference-sensitive condition” where shared decision-making is essential (37). However, Sitto et al. recently demonstrated that while both patients and physicians desire shared decision-making, this process is challenged by significant divergence in viewpoints regarding treatment priorities, education needs, and acceptable outcomes (29).

The synthesis also highlighted substantial unmet informational needs among women with OAB. Many participants reported confusion regarding the pathophysiology of OAB, available treatment options, expected outcomes, and long-term prognosis. Inadequate understanding often contributed to unrealistic expectations, disappointment, and frustration when treatments did not provide rapid or complete symptom resolution. At the same time, participants expressed a strong desire for autonomy, collaborative decision-making, and individualized therapeutic support. These findings support the integration of patient education and shared decision-making frameworks into OAB management pathways (38, 39).

Psychosocial burden emerged as another dominant aspect of treatment experiences. Embarrassment, stigma, concealment of symptoms, and fear of social judgment were pervasive across studies and frequently delayed help-seeking behavior. OAB also affected interpersonal relationships, occupational functioning, caregiving responsibilities, and emotional wellbeing. Importantly, caregivers and HCPs themselves described feelings of helplessness, frustration, and therapeutic uncertainty, suggesting that the burden of OAB extends beyond patients alone. These findings reinforce the need to conceptualize OAB as a biopsychosocial condition rather than solely a urinary disorder (40, 41).

Finally, this synthesis identified a major strain between the desire for rapid symptom relief and the sustained effort required for long-term behavioral management. Many participants favored pharmacological therapies perceived as more convenient or immediate, while behavioral interventions were frequently viewed as demanding, disruptive, or difficult to maintain. These findings may partly explain the limited long-term adherence to conservative therapies observed in clinical practice and highlight the importance of individualized treatment planning that considers patient preferences, daily routines, motivation, and perceived treatment burden. Recent reviews of patient preferences in OAB confirm this pattern, showing that patients consistently favor oral medications over behavioral or invasive therapies due to convenience, while also prioritizing assured benefits and avoiding side effects such as cognitive decline (10, 37). The convenience and empowerment associated with self-managed therapies has been highlighted as a key facilitator of adherence (25).

### 4.2 Strengths and limitations

The present synthesis has several strengths. To our knowledge, this is the first meta-synthesis to integrate treatment-related experiences and perceptions across the three principal stakeholder groups involved in OAB care: women with OAB, family caregivers, and HCPs. By synthesizing these complementary perspectives, the review moves beyond patient experiences alone to identify areas of convergence and divergence in treatment expectations, communication, and decision-making. This broader perspective provides a more comprehensive understanding of the factors influencing treatment engagement and may better inform patient-centred care pathways than evidence derived from a single stakeholder group.

The use of thematic synthesis allowed the integration of diverse qualitative findings into a higher-order interpretive framework capable of informing patient-centered clinical care. Methodological rigor was strengthened through independent screening, line-by-line coding by three researchers, iterative consensus discussions, reflexive practices, and structured quality appraisal using the CASP checklist (20) and transparent evaluation of confidence in findings using the GRADE-CERQual assessment (22).

Several limitations should also be acknowledged. First, only English-language studies were included, which may have limited the cultural diversity of perspectives represented in the synthesis. Moreover, the restriction to peer-reviewed publications may have excluded relevant qualitative evidence available in grey literature. Second, some included studies lacked detailed reporting regarding reflexivity, recruitment strategy, or ethical considerations, resulting in several CASP items being insufficiently detailed. Third, heterogeneity in study populations, healthcare settings, and investigated treatment modalities may have influenced the transferability of findings across healthcare systems. In addition, most studies originated from high-income countries, particularly the United States, the United Kingdom, and Hong Kong, potentially limiting applicability to lower-resource settings. An additional limitation is that four of the included studies originated from the same research group in Hong Kong. While these studies explored different stakeholder perspectives, including women with OAB, family caregivers, and primary care physicians, and addressed distinct research questions, similarities in methodological and interpretive approaches may have influenced the prominence of certain concepts within the synthesis. However, the principal analytical themes were consistently supported by evidence from independent studies conducted in other countries and healthcare settings, suggesting that the overall findings are robust despite this potential source of bias. Finally, as with all qualitative syntheses, interpretation of findings may have been influenced by the clinical backgrounds of the research team, although reflexive strategies and independent coding procedures were employed to minimize interpretive bias. Regarding the confidence levels of findings resulted after applying GRADE-CERQual assessment, four of the five review findings presented moderate confidence, considered to be reasonable representations of the phenomena of interest, although some concerns were identified across the CERQual components of methodological limitations, coherence, adequacy of data and relevance.

### 4.3 Interpretation of findings and future directions

The findings of this synthesis have important implications for clinical practice and are broadly aligned with contemporary international guidelines for OAB management, including those of the National Institute for Health and Care Excellence (NICE) (42), the European Association of Urology (EAU) (43), and the American Urological Association/Society of Urodynamics, Female Pelvic Medicine and Urogenital Reconstruction (AUA/SUFU) (38). These guidelines advocate patient education, shared decision-making, behavioural interventions as first-line treatment, and individualized management. The present synthesis complements these recommendations by providing qualitative evidence explaining why these principles are essential from the perspectives of patients, caregivers, and HCPs. Participants consistently described unmet informational needs, unrealistic treatment expectations, concerns regarding treatment burden, and the importance of therapeutic relationships, highlighting that successful long-term management depends not only on treatment efficacy but also on communication, continuity of care, and individualized support. The identified need for improved information, communication, and treatment support suggests that structured education and enhanced multidisciplinary support may warrant further evaluation. Digital symptom-monitoring or reminder tools could also represent potential approaches to supporting treatment engagement, although these strategies were not directly evaluated in the included qualitative evidence.

Future research should explore treatment experiences across more diverse cultural and healthcare settings, as well as among under-represented patient populations. Comparative qualitative studies examining differences according to age, menopausal status, treatment modality, symptom severity, healthcare system, or coexisting pelvic floor disorders may provide greater insight into the heterogeneity of OAB experiences and treatment perceptions. Additional research focusing on long-term treatment adherence and evaluating interventions designed to improve communication, expectation management, and behavioural support would further inform the development of personalised, patient-centred care pathways.

Finally, the analytical themes identified in this synthesis provide a preliminary conceptual framework highlighting aspects of OAB treatment experience that may warrant consideration in future patient-centred outcome-set and guideline development. These include quality of life, treatment burden, psychosocial well-being, therapeutic relationships, and patient engagement. However, the present synthesis does not establish these concepts as core outcomes or guideline recommendations. Their potential relevance and applicability require further development and validation through appropriate stakeholder consensus and methodological processes.

## 5 Conclusions

Women's experiences of OAB treatment are shaped by a complex interaction between symptom burden, treatment acceptability, healthcare relationships, psychosocial distress, and the desire for autonomy in care. This meta-synthesis highlights the need to consider dimensions beyond symptom control, including individualized communication, shared decision-making, realistic expectation setting, and recognition of the emotional and social impact of OAB. These findings support a more holistic and patient-centred approach to OAB management and identify aspects of care that warrant further evaluation in relation to treatment engagement, satisfaction, and long-term adherence.

## Data Availability

The original contributions presented in the study are included in the article/Supplementary Material, further inquiries can be directed to the corresponding author/s.
