# Supplementary material for: Perceptions of overactive bladder treatments among women, caregivers, and healthcare professionals: a systematic review and meta-synthesis
Source: Front Glob Womens Health. 2026 Oct 28;7:1974337. doi: 10.3389/fgwh.2026.1974337 (PMC13751609; doi:10.3389/fgwh.2026.1974337)
Supplement: Supplementary file 1 [file Table1.docx]

**Supplementary material 1:** Enhancing transparency in reporting the synthesis of qualitative research: the ENTREQ checklist

| **No** | **Item** | **Guide and description** | **Page(s)** |
| --- | --- | --- | --- |
| **1** | Aim | State the research question the synthesis addresses. | 2-3 |
| **2** | Synthesis methodology | Identify the synthesis methodology or theoretical framework which underpins the synthesis, and describe the rationale for choice of methodology *(e.g. meta-ethnography, thematic synthesis, critical interpretive synthesis, grounded theory synthesis, realist synthesis, meta-aggregation, meta-study, framework synthesis).* | 6-7 |
| **3** | Approach to searching | Indicate whether the search was pre-planned (*comprehensive search strategies to seek all available studies)* or iterative (*to seek all available concepts until they theoretical saturation is achieved)*. | 4-5 |
| **4** | Inclusion criteria | Specify the inclusion/exclusion criteria *(e.g. in terms of population, language, year limits, type of publication, study type).* | 4 |
| **5** | Data sources | Describe the information sources used (e.g. *electronic databases (MEDLINE, EMBASE, CINAHL, psycINFO, Econlit), grey literature databases (digital thesis, policy reports), relevant organisational websites, experts, information specialists, generic web searches (Google Scholar) hand searching, reference lists)* and when the searches conducted; provide the rationale for using the data sources. | 4 |
| **6** | Electronic Search strategy | Describe the literature search *(e.g. provide electronic search strategies with population terms, clinical or health topic terms, experiential or social phenomena related terms, filters for qualitative research, and search limits)*. | 4 |
| **7** | Study screening methods | Describe the process of study screening and sifting *(e.g. title, abstract and full text review, number of independent reviewers who screened studies).* | 4 |
| **8** | Study characteristics | Present the characteristics of the included studies *(e.g. year of publication, country, population, number of participants, data collection, methodology, analysis, research questions).* | 6-8 |
| **9** | Study selection results | Identify the number of studies screened and provide reasons for study exclusion *(e,g, for comprehensive searching, provide numbers of studies screened and reasons for exclusion indicated in a figure/flowchart; for iterative searching describe reasons for study exclusion and inclusion based on modifications t the research question and/or contribution to theory development).* | 5-6 |
| **10** | Rationale for appraisal | Describe the rationale and approach used to appraise the included studies or selected findings *(e.g. assessment of conduct (validity and robustness), assessment of reporting (transparency), assessment of content and utility of the findings).* | 6 |
| **11** | Appraisal items | [State the tools, frameworks and criteria used to appraise the studies or selected findings (e.g. Existing tools: CASP, QARI, COREQ, Mays and Pope [25]; reviewer developed tools; describe the domains assessed: research team, study design, data analysis and interpretations, reporting).](https://pmc.ncbi.nlm.nih.gov/articles/PMC3552766/#B25) | 6 |
| **12** | Appraisal process | Indicate whether the appraisal was conducted independently by more than one reviewer and if consensus was required. | 4-5 |
| **13** | Appraisal results | Present results of the quality assessment indicate which articles, if any, were weighted/excluded based on the assessment and give the rationale. | 13-14 |
| **14** | Data extraction | Indicate which sections of the primary studies were analysed and how were the data extracted from the primary studies? *(e.g. all text under the headings “results /conclusions” were extracted electronically and entered into a computer software).* | 5 |
| **15** | Software | State the computer software used, if any. | 5 |
| **16** | Number of reviewers | Identify who was involved in coding and analysis. | 5 |
| **17** | Coding | Describe the process for coding of data *(e.g. line by line coding to search for concepts).* | 5 |
| **18** | Study comparison | Describe how were comparisons made within and across studies *(e.g. subsequent studies were coded into pre-existing concepts, and new concepts were created when deemed necessary).* | 5-6 |
| **19** | Derivation of themes | Explain whether the process of deriving the themes or constructs was inductive or deductive. | 5 |
| **20** | Quotations | Provide quotations from the primary studies to illustrate themes/constructs, and identify whether the quotations were participant quotations of the author’s interpretation. | 13 |
| **21** | Synthesis output | Present rich, compelling and useful results that go beyond a summary of the primary studies (e.g. *new interpretation, models of evidence, conceptual models, analytical framework, development of a new theory or construct).* | 10-13 |

**Supplementary material 2:** Search query

( ( ‘’Refractory overactive bladder’’ ) AND ( ‘’Barriers’’ OR ‘’facilitators’’ OR ‘’Patient-centered’’ OR ‘’patient view’’ OR ‘’patient perspective’’ OR ‘’women perspective’’ OR ‘’lived experience’’ OR ‘’attitude OR ‘’belief OR narrative’’ OR ‘’phenomenology’’ OR ‘’grounded theory’’ OR ‘’thematic analysis’’ OR ‘’thematic synthesis’’ OR ‘’interpretative analysis’’ OR ‘’interview’’ OR ‘’focus group’’ OR ‘’ethnography’’ OR ‘’Phenomenological Analysis’’ OR ‘’reflexivity’’ OR ‘’qualitative study’’ OR ‘’qualitative research’’) AND ( ‘’botulinum toxin OR ‘’botox’’ OR ‘’onabotulinumtoxinA’’ OR ‘’BONTA’’ OR ‘’Percutaneous tibial nerve stimulation’’ OR ‘’PTNS’’ OR ‘’transcutaneous tibial nerve stimulation’’ OR ‘’sacral neuromodulation’’ OR ‘’SNS OR ‘’sacral nerve stimulation’’ OR ‘’SNM OR ‘’BTX’’ OR ‘’third line treatment’’ ) ).

**Supplementary material 3**: Modified Cochrane collection form

| **Data extraction field** | **Information extracted** |
| --- | --- |
| Study title | Title |
| Study ID (surname of first author and year first full report of study was published) | 1^st^ author’s surname, year of publication |
| Date form completed | (dd/mm/yyyy) |
| Context and participants | Population description  Setting (including location / social context)  Inclusion and exclusion criteria |
| Study design and methods used | Methodological approach of the study  Specific data collection and analysis methods utilized  Any theoretical models used to interpret or contextualize the findings  The data extraction approach and methods (interview, focus groups, observations, document analysis etc.) |
| Findings | Key themes / concepts identified in the primary studies.  Verbatim quotations of patients  Author’s interpretation of findings |
| Quality of the study | Critical Appraisal Skills Programme (CASP) for qualitative studies |

Supplementary material 4. Summary of analytical themes, contributing codes, and illustrative participant quotations

|  | **Contributing codes** | **Quotations from contributing studies** |
| --- | --- | --- |
| **Theme 1:**  **The burden of treatment versus the burden of illness** | Treatment side effects | “Yeah, it’s almost as if I caught all of the bad side effects. It ain’t worth it. It ain’t worth side effects,” ^24^ / "If it is necessary to take the medication, then it should be taken as long as there are no noticeable side effects" ^21^ / "… are the side effects worth the actual treatment? Or is the treatment worse than the actual side effects?" ^27^ |
|  | Treatment fatigue | “I’m just trying to get this done and over with as it is." ^24^ / "I have lost my hope to get cured. I am tired of the treatment, and maybe I will give up one day." ^30^ / “No remedy can offer me a cure. I feel very tired, and do not want to do any more treatment now" ^28^ |
|  | Treatment discontinuation | “I was taking the pills, and no, it didn’t meet my expectations at all. Actually I stopped it because it didn’t seem to be helping that much.’’ ^22^ |
|  | Aversion to medications | “I am real leery about taking medicine anyways” ^27^ /“If I did this one specific exercise, and that would help in the long run, then I’d rather do that. Something more natural, more organic, as opposed to taking more medicine.” ^27^ |
|  | Aversion to invasive procedures | “I didn’t want surgery. I didn’t want stuff up in me. I didn’t know what the side effects were.” ^24^ / “It seems that lately everybody wants to experiment and stuff and I’m just not up for all that poking and prodding, sticking, and doing all this different stuff.” ^24^ / I went to one doctor and they wanted to put needles in my back […] I said, ‘No way! You’re not putting any needles in my back.” ^24^ |
|  | Intimidating treatment setting | “It just started to terrify me. Just being in the theatre itself, people just being there you know I didn’t like that at all.” ^26^ / “The first time it was in the theatre and the second time it was down in the new hospital in a clinic room. I preferred the clinic room it seemed less intimidating” ^26^ /“ I was quite nervous really, cos it was quite dramatic being awake in somewhere in a place where you have an operation” ^26^ |
|  | Ineffective interventions | “All treatment fails to work on me.” ^26^ / “… she was dissatisfied because she failed to get better after several rounds of treatment that I had ordered.” ^29^ / “Basically, I don’t really feel like I’ve got anywhere.” ^25^ / “Doctors cannot help me much. I have been taking Western [biomedical] medications for several years but my problem still cannot get much improvement” ^28^ / “My bladder does not get any better, and the treatment is endless, painful, and strenuous” ^30^. |
|  | Lifestyle disruption | “It controls my life.” ^22^ / “It [OAB] definitely interferes […], if I’m not here I am in the bathroom.” ^22^ / “Don’t ever pass a toilet without stopping.” ^22^ / “Say cutting out caffeine entirely would help my symptoms and they would completely eliminate my symptoms. I don’t know if I’d be willing to do that. […] I would be absolutely miserable” ^27^. |
|  | Constant preoccupation and planning | “Going on car trips, long car trips, airplane trips. I mean it doesn’t stop me but you have to think where am I gonna sit, where am I gonna stop, where’s the next bathroom.” ^22^ /“ Even though I’m on those tablets, I’m still feeling like my life is ruled by wanting to go to the toilet all the time.” ^25^ / “It doesn’t really leave your mind. You’re sort of planning and thinking” ^25^. |
|  | Reduced quality of life | “I’m not enjoying my life to the fullest capacity” ^23^ / “I just reassess my lifestyle and see what can make it worse or what can make it better em, so … It’s just reassessing maybe my lifestyle, you know change it, just to make it better, hmm, to make my condition better uh to make it more … manageable” ^25^. |
|  | Lifelong burden | “Erm… it’s just you know… the bladder leakage. It’s just a part of life. It has been for a long, long time.” ^23^ / “There is no hope to get a cure, but the treatment is endless and the outcome is disappointing.” ^30^ |
|  | Desperation for symptom relief | “I’m tired of hurting. I just want the pain to stop for a minute.” ^24^ / “I was at the point where I would have done anything to get relief from the problem and I have nothing to lose by going on these trials”^26^ / “We are so desperate we will try anything.”^21^ / “Well, I’m willing to try anything to stop this.” ^23^ |
|  | Positive treatment outcome | “Yeah I would honestly say that my bladder was the best that it’s been for a long, long time” ^26^ / “…it took about three weeks then boom I stopped going to the toilet as much and I realised that I was going less frequently.” ^26^ / “so I now use the machine in the house, I’ve bought it and that’s keeping my symptoms at bay” ^23^ / “I could manage to get to the toilet in time with- out having to panic about it.” ^23^ |
| **Theme 2:**  **Erosion of trust in the therapeutic alliance** | Communication barriers | “First of all, a big one is that I have rapport with the doctor. That is number one. That I believe that they’re listening to me.” ^27^ / “My wife is unwilling to talk much about her problem; therefore, I dare not ask her about her condition.”^31^/ “Without knowing her needs and feelings, it is difficult for me to help her. However, I have no way of knowing because she refuses to talk about it.” ^31^ |
|  | Disappointment with healthcare | “Disappointed, so I assumed that I had been placebo although I may not have been. Although I think it was thinking about it.” ^26^ / “It was like going through all of that for nothing. I was kind of let down.” ^24^ |
|  | HCP limitations | “Some doctors asked me to see other doctors because they could find nothing wrong and so they could not help.”^28^ /“I suspected she might have OAB, but I could not confirm with confidence since I had my limitation [as a GP].”^29^ / “My understanding is limited, I will tell you that.”^33^ /“ Therefore, there was little more that I could do except advise her to relax” ^29^ |
|  | Diagnostic challenge | “When I first experienced from the symptoms, I had been shopping different doctors and had spent many years to get diagnosed” ^30^ / “I guess the big challenge is just getting them to admit it.” ^33^ / “I guess some of them just being upfront and honest and letting me know. If say for some reason I forget to ask them, they don’t think about it, you know, then just getting our patients to let me know what their problem is.” ^33^ |
|  | Mistrust towards HCPs | “Some patients indeed feel offended about such a question, particularly those patients who have seen me for many years, and this can ruin the trust between us” ^29^ / “… she refused, and accused me of cheating her out of money by prolonging treatment and referring her to another specialist.” ^29^ / “I blamed my hysterectomy, I say [my gynecologist] did something to my bladder”^32^ |
|  | Skepticism | “No surgery and no medication can treat it. The treatment often is useless and unable to relieve the symptoms much” ^30^/ “I’ve heard [of] Botox. I don’t think I would want to try that. Because people use it for cosmetic stuff.” ^24^ |
|  | Prescribing pressure | “I finally gave her a course of antibiotics to satisfy her. By prescribing antibiotics, you can make the patient trust you, although you know that you have limitations in dealing with the patient’s condition.” ^29^ / “I usually prescribe a course of antibiotics to make them satisfied, even though I know antibiotics have nothing to do with their condition.” ^29^ |
|  | Treatment expectations (when unmet) | “The goal isn’t always to make things 100 % perfect, it’s just to make things more liveable and more so the symptoms aren’t as bothersome.” ^27^ / “They often expect quick efficacy after a course of medication or two, although such an outcome is ‘mission impossible’ in their cases.” ^29^ / “First you go to the doctor for advice and if that doesn’t work you start playing doctor yourself doing what you think should be done. I mean what can you do?” ^32^ |
| **Theme 3:**  **The knowledge gap and desire for agency** | Lack of knowledge | “Being aware of more symptoms and asking, and treatment options were really good. I had no idea they put Botox. I mean, I had no idea they did that.” ^33^ / “I know that he stapled hers so I was gonna go for what she had, because I know what Botox is and I don’t know if I want that in my bladder or even what it’s gonna do. But I understand staples.” ^24^ |
|  | Desire for information | “First you go to the doctor for advice and if that doesn’t work you start playing doctor yourself doing what you think should be done. I mean what can you do?“ ^22^ / “I should have gotten in there and read more. Instead of relying so much on the doctor. You know, it was like, if he said jump, I would jump. I should have taken matters into my own hands, maybe, and gotten through this process faster.” ^27^ |
|  | Symptom awareness | “I didn’t realise, but I was maybe getting up two or three times in the night.” ^25^ / “It’s just that I’ve got a weak bladder, you know. But now I’m conscious of it all the time.” ^25^ / “I’ve seemed to have noticed it more the last couple of years cos I moved in with my [family].” ^25^ |
|  | Decision making | “And looking more at how other people combine medications and things like that is always reassuring, and looking at different options.” ^33^ / “Honestly, I was referring a little too fast. I definitely need to try the dual therapy” ^25^ / “We say all the options are appropriate for someone, but usually I think depending on the symptoms the patient has and the clinical history, I don’t think all the options are equally good for any patients. There’s usually one option that I think it’s a little bit better.” ^27^ |
|  | Individualized treatment support | “Then you can have a little bit of a higher-level conversation because they already know what the different treatment options are and you can really get into their preferences a little bit more.” ^27^ / “You plug in their frequency, their age, their quality-of-life scores and then tell them what responses they might expect from different therapies…” ^27^ |
|  | Openness | “I mean, the key word again is never say never,” ^24^ / “Well I was willing to give anything a try” ^26^ / “Well I felt positive well I thought we’ll give it a go it can’t be any worse than what’s going on at the moment so yes it was I was glad that I was going to take part in it and to see if something was going to work.” ^26^ |
|  | Treatment expectations | “I had some physiotherapy at the hospital for a couple of months, 3 months, I didn’t think it would help them really because my muscles were shall we say too slack” ^26^ /“… this is why I went to see the GP in the very very first place, I just would like to get a full night’s sleep. That’s basically all I’ve really wanted out of it.” ^25^ |
|  | Desire for immediate results | “Usually patients expect to be treated quickly when they come to a doctor. If they fail to recover quickly, they blame the doctors either for being incapable or deliberately prolonging treatment” ^29^ / “This didn’t happen overnight, but we’re supposed to fix it overnight.” ^33^ |
|  | Commitment to treatment | “Well it was very clear it was something I didn’t find easy but it was very clearly put to me. It was not something I enjoyed but I was pleased that if I needed to do I was able to do” ^26^ /“ It was a little bit uncomfortable but not to the point where I wouldn’t have it done again under the local if I went back a second time” ^26^ |
|  | Acceptance of diagnosis | “After all, the disease itself will not kill you even if you leave the symptoms untreated. I have been thinking about giving up the treatment, but [it is] just that I am still struggling.” ^28^ / “A way of life, yeah.” ^28^ |
|  | Optimism | “I thought if anything helps then go for it, if it’s going to change my lifestyle. So I was up for that.” ^26^ / “The second set they said I would definitely get it the second time and I was in one way pleased I knew it hadn’t worked and it wasn’t the real thing (placebo). I was hopeful that the second set of injections that it was going to be effective that it was going to work.” ^26^ |
| **Theme 4:**  **Psychosocial barriers to treatment engagement** | Embarrassment | “But the thing is, I told him that I get embarrassed. I get like I’m disabled or something” ^24^ / “I cannot tell my friends the truth, because they are just my friends. I do not want to embarrass my wife; bladder problems are really embarrassing.” ^31^ |
|  | Stigma | “I do not want others know about my bladder problem, because I am afraid that they will label me as taking ''little K'' [ketamine]” ^30^ /“Such negative label has been deeply rooted in people's mind now. Keeping my situation in secret should allow me to avoid troubles” ^30^ |
|  | Social isolation | “They avoid having lunch with me and avoid working with me.” ^30^ /“ I rarely hang out with my friends now, because I do not know how they will think of me.” ^30^ / “I just stay at home unless necessary, because I really feel very anxious about going out.” ^28^ |
|  | Helplessness | “I wish I could do more, but I do not know what I can do to help her feel better.” ^31^ / “I feel so helpless because even my family members cannot understand my difficulty” ^28^ / “There is no hope to get a cure, but the treatment is endless and the outcome is disappointing.” ^30^ |
|  | Psychological distress | “He just cannot understand how this disease can bring such a lot of psychological pressure to me.” ^28^ / “It was absolutely horrendous it was taking over my life because I was in a really sort of dark place with it, it was dreadful and it was just ruling my life.” ^26^ |
|  | Frustration | “I am really very frustrated. I have received all treatment and obeyed what the doctors said, but the situation just keeps going worse and worse. I really cannot accept it.” ^28^ / “However, my situation fails to get any better even [though] I have received treatment for many years. I really feel very frustrated. What else can I do” ^28^ |
|  | Affected relationships | “My husband cannot understand much about my difficulty.” ^28^ |
|  | Impact on professional life | “My boss has warned me several times that going to toilet often will reduce the company's productivity, and he will fire those who be- have against the law.” ^30^ |
|  | Caregiver burden | “Every time I see my wife suffering from the disease, I feel guilty because I cannot find a good doctor and a good remedy to treat her.”^31^ / “Because of the disease, my wife’s mood is bad and she always loses her temper. I feel very bad because I am unable to help her.” ^31^ |
|  | Reduced quality of life | “I’m not enjoying my life to the fullest capacity” ^23^ / I just reassess my lifestyle and see what can make it worse or what can make it better” ^25^ |
| **Theme 5:**  **Preference for low-burden treatments and resistance to behavioural change** | Treatment convenience | “Well they’re portable, they’re lightweight, they don’t use up a lot of energy.... there’s not really anything that would stop me from using it again” ^23^ / “As I said, it was really easy to do, I’ve got to say, it was literally just sticking the pads on and sticking it on, and just turning it up.” ^23^ |
|  | Preference for medication over behavioural therapy | “They don’t want to do the work. Bladder diaries are not fun, and bladder training and time voiding. They’re like, just give me a pill.” ^33^ / “…well, just give me the pill now and I won’t have to do that stuff.” ^33^ |
|  | Lifestyle modifications | “If I try and just limit my toilets and drinks I’m not too bad” ^25^ / “I try not to drink coffee after noon” ^22^ / “It was a nuisance my quality of life was being affected you had to be aware; all the time where toilets were, wear protection all the time and occasionally you were embarrassed.” ^26^ |
|  | Treatment barriers | “Just ’cause I don’t have money, doesn’t mean I should be wet all the time.” ^24^ /“ I think those things [pelvic floor physical therapy] you have to do consistently, and I have four kids and I work so consistently isn’t quite easy yet.” ^27^ / “… would [start third-line therapy right away] if I could. I’m limited by insurance companies.” ^27^ |
|  | Commitment to treatment | “Just because you’ve (a) bladder problem doesn’t mean your life stops.” ^23^ |
|  | Desire for immediate results | “They’re like, just give me a pill.”^33^ / “I know what everyone would like is to come in, take one pill, which I actually give them in the office, and be cured.” ^33^ |
|  | Seeking alternative options | “I have searched for many different remedies – western medicine, Chinese medicine, natural therapies and even folk remedies – for my sister to try.” ^31^ / “I also follow behavioral therapy, and change my habit of liquid consumption and voiding as much as I can.” ^28^ |
|  | Skepticism | “[…] unsure whether these tablets worked or not”^20^ / “I cannot see why I still need to keep on with the treatment if the treatment cannot help me.” ^30^ / “No surgery and no medication can treat it. The treatment often is useless and unable to relieve the symptoms much.” ^30^ |
|  | Acceptance of diagnosis | “It’s just learning and living with the condition isn’t it?” ^25^ |
|  | Optimism | “I do not think that incontinence will follow me for the rest of my life.” ^30^ / “I am still young, so I do not think I will lose control of my bladder forever.” ^30^ / “These symptoms will not last for long; they are just temporary, I think.” ^30^ |
